# Provider- and Patient-Related Barriers to and Facilitators of Digital Health Technology Adoption for Hypertension Management: Scoping Review

**DOI:** 10.2196/11951

**Published:** 2019-03-26

**Authors:** Ramya Sita Palacholla, Nils Fischer, Amanda Coleman, Stephen Agboola, Katherine Kirley, Jennifer Felsted, Chelsea Katz, Stacy Lloyd, Kamal Jethwani

**Affiliations:** 1 Partners HealthCare Pivot Labs Boston, MA United States; 2 Harvard Medical School Boston, MA United States; 3 Massachusetts General Hospital Boston, MA United States; 4 American Medical Association Chicago, IL United States

**Keywords:** medical informatics, culturally appropriate technology, hypertension

## Abstract

**Background:**

The uptake of digital health technology (DHT) has been surprisingly low in clinical practice. Despite showing great promise to improve patient outcomes and disease management, there is limited information on the factors that contribute to the limited adoption of DHT, particularly for hypertension management.

**Objective:**

This scoping review provides a comprehensive summary of barriers to and facilitators of DHT adoption for hypertension management reported in the published literature with a focus on provider- and patient-related barriers and facilitators.

**Methods:**

This review followed the methodological framework developed by Arskey and O’Malley. Systematic literature searches were conducted on PubMed or Medical Literature Analysis and Retrieval System Online, Cumulative Index to Nursing and Allied Health Literature, and Excerpta Medica database. Articles that reported on barriers to and/or facilitators of digital health adoption for hypertension management published in English between 2008 and 2017 were eligible. Studies not reporting on barriers or facilitators to DHT adoption for management of hypertension were excluded. A total of 2299 articles were identified based on the above criteria after removing duplicates, and they were assessed for eligibility. Of these, 2165 references did not meet the inclusion criteria. After assessing 134 studies in full text, 98 studies were excluded (full texts were either unavailable or studies did not fulfill the inclusion criteria), resulting in a final set of 32 articles. In addition, 4 handpicked articles were also included in the review, making it a total of 36 studies.

**Results:**

A total of 36 studies were selected for data extraction after abstract and full-text screening by 2 independent reviewers. All conflicts were resolved by a third reviewer. Thematic analysis was conducted to identify major themes pertaining to barriers and facilitators of DHT from both provider and patient perspectives. The key facilitators of DHT adoption by physicians that were identified include ease of integration with clinical workflow, improvement in patient outcomes, and technology usability and technical support. Technology usability and timely technical support improved self-management and patient experience, and positive impact on patient-provider communication were most frequently reported facilitators for patients. Barriers to use of DHTs reported by physicians include lack of integration with clinical workflow, lack of validation of technology, and lack of technology usability and technical support. Finally, lack of technology usability and technical support, interference with patient-provider relationship, and lack of validation of technology were the most commonly reported barriers by patients.

**Conclusions:**

Findings suggest the settings and context in which DHTs are implemented and individuals involved in implementation influence adoption. Finally, to fully realize the potential of digitally enabled hypertension management, there is a greater need to validate these technologies to provide patients and providers with reliable and accurate information on both clinical outcomes and cost effectiveness.

## Introduction

Digital health technologies (DHTs) have the potential to support active self-management of chronic conditions via education, monitoring and support, timely feedback, and remote access to health professionals [1]. When designed and implemented successfully, digital health interventions offer an opportunity to support the quadruple aim of health care by improving health outcomes, increasing patient experience, reducing health care costs, and improving clinician satisfaction [2]. The American Medical Association (AMA) defines digital health tools as those systems and solutions that engage patients for clinical purposes, collect, organize, interpret, use clinical data, and manage outcomes and other measures of care quality including telemedicine and telehealth, mobile health, wearables, remote monitoring, and apps [3]. The AMA digital health survey classifies digital health solutions into 7 categories: remote monitoring for efficiency, remote monitoring and management for improved care, clinical decision support, patient engagement, televisits, point-of-care, and tools providing consumer access to clinical data [3].

One-third of the US population has hypertension (85.7 million adults) [4] and the economic burden is close to US $ 53 billion dollars annually [5]. Despite having access to effective drugs for lowering blood pressure (BP), BP control in a vast majority of patients remains suboptimal [5], owing to infrequent monitoring of BP [6], low medication adherence by patients [7], and clinical inertia [8]. DHTs for hypertension management, such as telemonitoring programs, enhance self-monitoring as they allow for BP readings and clinical information to be shared with health care professionals in real time [9]. Remote monitoring for hypertension has been shown to improve medication adherence [10], optimize BP control [11], and reduce use of health care resources [12].

Although the shift to a value-based care system has encouraged the adoption and use of DHT to manage hypertension, the uptake of DHTs has been surprisingly low in clinical practice [13]. In addition, to our knowledge, there is limited information on the factors that influence adoption of digital health from the perspectives of both patients and providers. Previously published literature includes surveys of providers that cite factors influencing DHT adoption such as organizational and financial barriers [14]. Previous systematic reviews of telemedicine for hypertension management report increased access to health services, improved health and quality outcomes, and enhanced patient knowledge and involvement in disease management as strong facilitators of DHT usage in health care settings [13,15]. This review provides a comprehensive summary of facilitators and barriers to adopting digital health for hypertension management with a specific focus on the perspectives of providers and patients.

## Methods

### Literature Search

This scoping review was conducted using the methodological framework developed by Arskey and O’Malley [16]. The Arksey and O’Malley framework is particularly suited to address broad research questions and can help map the current literature, extract key concepts and themes, and identify gaps. The Arksey and O’Malley framework has several steps including (1) identifying the broad research question, (2) study selection using inclusion or exclusion criteria on the basis of familiarity with the topic of interest, (3) sorting the extracted data from studies into themes and patterns, and (4) collating key themes and issues [16]. The primary research question guiding this review was the following: *What are the barriers and facilitators of digital health adoption for hypertension management?*

Structured literature searches were conducted using 3 databases to identify relevant studies from 2008 to 2017: PubMed or Medical Literature Analysis and Retrieval System Online, Cumulative Index to Nursing and Allied Health Literature (CINAHL), and Excerpta Medica database (EMBASE). Medical subject headings (MeSH) and selected keywords were searched using Boolean operator *OR* and these groups were combined using another Boolean operator *AND*. Keywords used include (1) *hypertension* (MeSH), *hypertensi*, (2) *mobile applications* (MeSH), *mobile device*, (3) *electronic health records* (MeSH), *personal electronic health record*, (4) *decision support systems, decision support*, (5) *remote monitoring* (MeSH), (6) *providers* (MeSH)*, clinician*. The detailed search strategies for PubMed have been provided as an example (see Multimedia Appendix 1). At first, 2 reviewers, with subject matter and methodological expertise, independently reviewed all abstracts identified by the searches and conflicts were resolved by a third reviewer. Then, 2 reviewers screened the full texts to select the final studies to be included in the review. Cohen kappa test revealed an agreement score of 0.75 between the reviewers. Per Landis and Koch, this agreement score could be categorized as *substantial agreement* between the reviewers [17].

All articles retrieved were screened using the following inclusion criteria: (1) reported on adoption barriers and/or facilitators of digital health solutions, as defined by the AMA, that were provider- or patient-related, (2) focused on hypertension management, (3) published in English, and (4) published between 2008 and 2017. Studies were excluded if they (1) did not report on barriers or facilitators of digital health, (2) described barriers or facilitators exclusively for nonclinical staff such as pharmacists, (3) were editorials or reviews for editorials, epidemiological studies, and protocols, (4) provided insights on acute management of hypertension in perioperative or intensive care settings, or (5) if full texts were unavailable. The authors also conducted a gray literature search (including conference proceedings) through a Web search engine. In addition, 4 articles were handpicked on the basis of the same inclusion criteria used for articles selected via literature databases.

### Thematic Analysis

The selected papers were reviewed to extract relevant data. A data extraction template developed by the authors was used to extract key information and concepts from the included studies and the template included the following constructs: the geography, study design, program setting, disease conditions (in addition to hypertension), study objectives, sample description, sample size, digital health category, design features of the intervention, clinical outcomes, cost outcomes, patient experience, provider experience, patient-related barriers and facilitators, and provider-related barriers and facilitators. Descriptive and inductive thematic analyses were conducted for identifying major themes pertaining to barriers and facilitators of DHT adoption. For the analysis of the text passages from the included articles, the inductive thematic analysis was conducted as described by Braun and Clarke [18]. We developed our own a priori framework to categorize barriers into the following 4 categories: (1) provider-related facilitators, (2) provider-related barriers, (3) patient-related barriers, (4) and patient-related facilitators. This analytic process involved reading and rereading of the selected papers, systematically identifying and naming the unit of meaning with codes (words or sets of words that provide a meaning label), and then searching for patterns in the data and organizing the data (smaller themes or codes) into larger themes representing the main ideas and their relationships. Themes were then reviewed by the team and representative data elements were selected to demonstrate the salient themes. At first, 2 investigators (RP and NF) independently performed the initial coding of the first transcript. This coding was then reviewed by the third reviewer (AC). The codes were then reviewed and discussed with the team including senior researchers in the field, providers, and other subject matter experts. Later, 2 reviewers (RP and NF) then recoded all papers, integrating feedback from the team into the coding structure. A final codebook was created using Microsoft Office Excel (version 1808) on the basis of the consensus of the 3 investigators (RP, NF, and AC). During this process, any discrepancies in coding were discussed and resolved among all investigators. Furthermore, any questions about meaning and interpretation of themes were discussed among the team members and resolved through consensus.

## Results

### Overview

**Figure 1:**
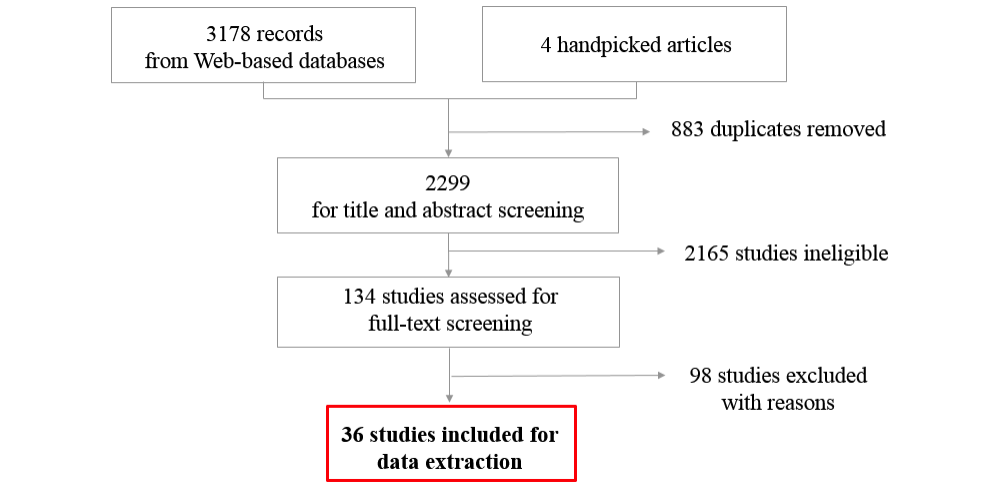
Study selection flow diagram.

A total of 2299 titles and abstracts from PubMed, CINAHL, EMBASE, and 4 handpicked articles from the supplementary gray literature search were assessed for eligibility after removing duplicates (see Figure 1). Of these, 2165 references did not meet the inclusion criteria. After assessing 134 studies in full text, 98 studies were excluded (full texts were either unavailable or studies did not fulfill the inclusion criteria). A total of 36 studies satisfied the inclusion criteria, including the 4 handpicked articles. The articles included in this review were published between 2008 and 2017, with a majority (n=30) published after 2010. Studies were published across the following countries: United States (n=21), United Kingdom (n=4), Canada (n=3), Finland (n=1), Sweden (n=1), Italy (n=1), Taiwan (n=1), Malaysia (n=1), South Korea (n=1), Kenya (n=1), and Germany (n=1). DHTs included in this review were classified into categories as defined by the AMA: remote monitoring for efficiency (n=6), remote monitoring and management for improved care (n=19), clinical decision support (n=6), patient engagement (n=4), televisits or virtual visits (n=6), point-of-care(n=2), and tools providing consumer access to clinical data (n=1). Most studies were conducted in a primary care setting (n=30). A plurality of studies included qualitative assessments (n=15). Quantitative methodologies included randomized controlled trials (RCTs; n=14), nonrandomized trials (n=2), usability pilots (n=2), and pre and poststudies (n=2). In addition, 1 white paper was also included in this review. Multimedia Appendix 2 displays a summary of the studies included in this review. The results of the thematic analysis have been categorized as provider- and patient-related facilitators and barriers as detailed below. Tables 1 and 2 summarize all the themes.

**Table 1:** Summary and frequency of provider-related themes and sub-themes identified from authors’ thematic analysis of the 36 studies in this review. Most studies included in the review reported multiple themes. Frequency of a barrier or a facilitator=total number of occurrences of a facilitator or the barrier and total frequency of occurrences of facilitators and barriers.

Variable		Occurrences and frequency, n (%)
**Facilitators^a^**		
	1. Ease of integration with clinical workflow [19-25]; Actionable data to provide timely interventions to patient [20,22,23,26]; Integration with clinical routine and less time-consuming tasks [20,21]; Care team support: opportunity for delegation and team-based care [19,20,24,25]	8 (33)
	2. Improvement in patient health outcomes [20,23,25,27,28]; Technology prevalidated to improve outcomes [20,25,27]; Positive impact on patients and their self-management [20,27,28]; Better monitoring of patients to prevent negative outcomes [23]	5 (21)
	3. Technology usability and technical support [29-36]; Technology requires minimal training [29,35,36]; Ease of use [29,30,35,36]; Adequate training support [31-33]	8 (33)
	4. Financial factors [27,37]	2 (8)
	5. Leadership and organizational support [38]	1 (4)	
**Barriers^b^**		
	1. Lack of integration with clinical workflow [9,19-21,24,25,30,39-41]; Lack of integration with electronic medical record [24]; Additional time-consuming tasks for providers [9,19-21,39-41]; Clinically irrelevant data [25,30]	10 (36)
	2. Lack of validation of technology [14,32,38,42-45]; Concern over data accuracy [14,42-44]; Lack of evidence of improvement in patient outcomes [32,38,45]	7 (25)
	3. Concern over data privacy and security [32]	1 (4)
	4. Lack of technology usability and technical support [30,34,38-40,43,46]; Frequent technical issues [34,39] Lack of ease of use [30,39,40,43,46]; Long learning curve [38]	7 (25)
	5. Lack of leadership and organizational support [32,40]	2 (7)
	6. Increased patient anxiety [14]	1 (4)

^a^Total frequency of occurrences of facilitators=20.

^b^Total frequency of occurrences of barriers=28.

**Table 2:** Summary and frequency of patient-related themes and sub-themes identified from authors’ thematic analysis of the 36 studies in this review. Most studies included in the review reported multiple themes. Frequency of barrier and facilitator=total number of occurrences of a facilitator or barrier and total frequency of occurrences of facilitators or barriers.

Variable		Occurrences and frequency, n (%)
**Facilitators^a^**			
	1. Technology usability [19,24,30,34,36,46-50]; Ease of use [19,24,30,36,46,48-50]; Technical support [47,48]; Integration into patient’s daily routine [46]	10 (29)
	2. Positive impact on patient-provider communication [19,20,28,37,46,49-51]; Improved and more timely feedback from providers [19,20,28,37,46,49-51]; Shared decision making with providers [46]; Better preparation for clinic visits [28]	8 (24)
	3. Improved self-management and patient experience [19,21,24,30,33,36,39,46,52]; Increased motivation to better manage health [36,39]; Increased access to health data [21,24,30,33,36,46]; Alleviation in anxiety from better monitoring of health data [19,52]	9 (26)
	4. Reduction of in-office visits [19,21,24,25,37,41,52]	7 (20)
**Barriers^b^**		
	1. Lack of technology usability and technical support [14,19,20,30,47,48]; Frequent technical glitches [14,19,21,50,53]; Lack of ease of use of system [43,50]; Patient not confident in using device [14,19,50]	9(41)
	2. Interference with patient-provider relationship [19,20,37,42,47]; Fear of having less direct in-person communication with provider [19,37]; Lack of feedback from providers [42,47]; Disrupting feelings of independence [20,37]	5 (23)
	3. Lack of validation of technology [19,43,47]	3 (14)
	4. Increased patient anxiety [49,52]	2 (9)
	5. Concern over data privacy and security [48]	1 (5)
	6. Cost of digital health equipment [42,47]	2 (9)

^a^Total frequency of occurrences of facilitators=34.

^b^Total number of occurrences of barriers=19.

### Facilitators of Digital Health Adoption

#### Provider Factors

##### Ease of Integration With Clinical Workflow

The findings suggest that integration of a new technology into the existing workflow of a provider strongly influences DHT adoption (n=2) [20,21]. Providers cited that having a care team to support DHT implementation as part of the clinical workflow was an important facilitator of adoption (n=4) [19,20,24,25]. Some studies found that providers were able to successfully adopt DHTs when the data that the DHT provided were actionable and could be readily utilized within preexisting clinical workflows to enable timeline intervention to improve patient outcomes (n=4) [20,22,23,26]. Providers were also attracted to DHTs that provided automatic alerts identifying the need for a change in medications or dosage [23], as they helped perform routine tasks faster (n=1).

##### Improvement in Patient Health Outcomes

Providers’ beliefs regarding whether the technology improved clinical outcomes or engaged patients in self-management were among the most important considerations (n=3) [20,27,28] for embracing DHTs. In some instances, the DHTs that were validated in pilot and RCTs and shown to improve outcomes were perceived to be more acceptable to providers (n=4) [20,25,27,28]. Furthermore, providers valued their patients becoming more active and engaged in their own health (n=2) [20,28]. Finally, DHTs that enabled a more timely response to elevated BP levels helped providers prevent adverse health outcomes in their patients by addressing the changes in BP levels in a timely manner (n=1) [23].

##### Technology Usability and Technical Support

Some studies reported that providers valued the simplicity and ease of use of a system (n=4) [29,30,35,36]. Furthermore, providers preferred DHTs that required minimal training (n=3) [29,35,36]. Providers valued adequate technical support when using DHTs as a part of their clinical workflow (n=3) [30,34,35].

##### Financial Factors

A few studies reported that financial incentives such as physician reimbursement for using DHTs in their clinical practice and cost savings as a result of implementing DHTs were important influencers of provider adoption (n=2) [27,37].

##### Leadership and Organizational Support

An organizational culture of innovation coupled with the presence of *physician champions* was cited as a factor influencing the adoption of DHTs in clinical settings, as it was often difficult for clinicians to implement DHTs without the support of their organization and leadership, particularly in terms of required budget and personnel (n=1) [38].

#### Patient Factors

##### Technology Usability and Technical Support

DHTs that were easy to use and included timely technical support [19,24,30,34,36,46,47,49,50] fostered patient engagement (n=9). Older patients and those with less experience using technology reported that technical support was a facilitator (n=2) [47,48]. Patients valued solutions that were easy to integrate into their daily routines (n=1) [46]. Interventions were more easily adopted when they were culturally tailored for specific target populations (n=1) [34].

##### Improved Patient-Provider Communication

Improved communication with providers was a facilitator of adoption for patients. Some patients reported that DHTs enabled direct contact with their providers to share their health data and receive feedback [19,20,28,37,46,49-51]. Data sharing via DHTs helped patients better understand their care plans and promoted shared decision making [46]. DHTs improved visit preparation and accuracy of patient-provided information [28].

##### Improved Self-Management and Patient Experience

Patients were more likely to adopt DHTs that increased their motivation to manage their own conditions (n=2) [36,39]. Patients reported that being able to access and view their health data from their own device encouraged them to be more proactive about their health (n=6) [21,24,30,33,36,46]. Several studies reported greater patient satisfaction using DHTs for hypertension management (n=6) [19,36,37,47,48,52]. Some patients found that using DHTs to monitor their BP readings helped alleviate health-related anxiety (n=2) [19,52].

##### Reduction of Office Visits

The opportunity for patients to potentially avoid having to travel to the physician’s office was reported as a facilitator of DHT adoption by patients in some studies (n=7) [19,21,24,25,37,41,52].

### Barriers for Digital Health Adoption

#### Provider Factors

##### Lack of Integration With Clinical Workflow

Several studies reported the lack of integration of technology with clinical workflow as a major barrier to DHT adoption (n=6) [21,24,25,39-41]. The lack of care team resources available to successfully implement DHTs and perform additional tasks was highlighted by multiple studies (n=3) [19,20,24]. Too many additional tasks associated with implementing DHTs were reported to be problematic for several providers (n=1) [9].

##### Lack of Validation of Technology

Some providers cited concerns over accuracy of data as a potential road block to using home BP monitors on a wider scale (n=4) [14,42-44]. Another barrier to provider adoption was the lack of evidence or proof that DHTs improved patient outcomes (n=3) [32,38,45].

##### Concern Over Data Privacy and Security

One study reported that the lack of assurance of patient data security was a big concern for providers as well (n=1) [32].

##### Lack of Technology Usability and Technical Support

Another barrier frequently highlighted in the literature was the complexity of technologies (n=5) [30,39,40,43,46]. Frequent technical issues coupled with inadequate onsite support to resolve them were cited as reasons for discontinuing engagement with DHTs (n=2) [34,39]. Furthermore, the learning curve associated with new DHTs made it difficult for providers to balance the use of a new system and keep up with their daily clinical routine (n=1) [38].

##### Lack of Organizational Support

Organizational factors, such as lack of leadership support for integrating technology in practice and budget constraints, delayed implementation of new DHTs (n=2) [32,40]. Hospital budgets were too constrained to gather additional resources necessary to implement DHTs as part of the clinical practice workflow (n=1) [32].

##### Increased Patient Anxiety

One study reported that providers were concerned that patients may be more anxious if they continuously monitored their BP data and believed excess data could be more harmful than useful for the patients (n=1) [14].

#### Patient Factors

##### Lack of Technology Usability and Technical Support

Technical issues such as password access, connectivity, and usability prevented patients from using DHTs (n=5) [14,19,20,30,48]. Patients often preferred DHTs that were easy to use regardless of technical skills and abilities and were less time consuming (n=2) [47,48]. Patients with impaired vision, low dexterity, and chronic conditions had difficulties adopting DHTs into their routine (n=3) [14,20,48].

##### Interference With Patient-Provider Relationship

Patients expressed concerns that using DHTs would interfere with their current in-person relationship with their providers (n=2) [19,37]. Another barrier that patients experienced was the lack of timely feedback from the provider when using DHTs with a provider-facing portal (n=2) [42,47]. In some cases, DHTs were viewed as an impediment to patients’ feelings of independence as they were forced to share data with providers they may not want to (n=2) [19,20].

##### Increased Patient Anxiety

Some patients experienced anxiety from using DHTs (n=2) [49,52]. This anxiety stemmed from checking their BP too often and being unable to contact their provider directly and obtain timely feedback (n=2) [49,52].

##### Concern Over Data Privacy and Security

Patients were comfortable with access to health data being limited to only themselves and their providers. However, patients were concerned about the privacy of data shared via DHTs and were uncomfortable with the risk of a third party accessing their data [48].

##### Lack of Validation of Technology

In some studies, patients questioned the accuracy of the measurements and data recorded (BP readings) by DHTs [19,43,47].

##### Cost of Digital Health Equipment

The cost of digital health equipment was also cited as a barrier to adoption [42,47]. Some patients also expressed concern over being liable for cost of damage to the equipment [47].

## Discussion

### Principal Findings

This review contributes to existing literature by highlighting factors that enable or hinder the adoption of digital health solutions from the perspectives of both providers and patients. These results show that the key facilitators of DHT adoption by physicians include integration with clinical workflow 33% (8/24), ease of use 21% (5/24), improvement in patient outcomes 21% (5/24), financial factors 8% (2/20), and organizational support 4% (1/20). Technology usability and technical support 29% (10/35), positive impact on well-being and self-management 26% (9/35), improved patient-provider relationship 24% (8/35), and a reduction of in-office visits 20% (7/35) were most frequently reported facilitators for patients. The most frequently reported barriers to use of DHTs reported by physicians include lack of integration with workflow 36% (10/28), lack of validation of technology 25% (7/28), and lack of usability and support 25% (7/28). Finally, a lack of technology usability 41% (9/22), interference with the patient-provider relationship 23% (5/22), and lack of validation of technology 14% (3/22) were the top barriers reported by patients.

Although these findings highlight some common themes reported in previous work, there are several key differences and contributions from this study. A 2017 study by Mileski et al, examining the facilitators and barriers to implementing telemedicine for hypertension management [13], only focused on telemedicine, whereas our study examined all DHTs from the perspective of both patients and providers. Consistent with Mileski et al, we found that improved outcomes, increased patient knowledge and self-management, and cost savings were important facilitators of DHT adoption. Another systematic literature review by Gagnon et al [15] evaluated the factors influencing adoption of DHTs by health care professionals and some barriers reported in this review, such as the lack of organizational support and lack of reimbursement for providers, these were consistent with our study findings. Furthermore, most of the studies included in the review by Gagnon et al were conducted in large hospitals. In contrast, most studies in our review, 86% (31 out of 36 studies), were conducted in primary care settings. Additionally, Gagnon et al [15] examined DHTs across multiple diseases, whereas our review focused specifically on DHTs for hypertension management.

Multiple conceptual models exist to describe acceptance and usage of technology, such as Rogers diffusion of innovations theory [54], the technology acceptance model [55], and the unified theory of acceptance and use of technology (UTAUT) [56]. These models have been applied to describe the adoption of electronic health records and other forms of DHTs [57]. As a thematic analysis approach was used to identify new or emergent themes, we neither tied our analysis to a preexisting conceptual model nor sought to validate a preexisting conceptual model. However, it is worth noting that the themes that emerged from our analysis align with several of the constructs described in UTAUT. For example, the themes of clinical workflow integration and technology usability relate to the UTAUT construct of effort expectancy. Similarly, the theme of improvement in patient outcomes relates to the UTAUT construct of performance expectancy.

### Future Implications

Lack of usability or ease of use was found to be a major barrier for both patients and providers in our review. Furthermore, lack of integration with clinical workflow was an important barrier for physicians. In the light of these findings, it is important that developers of DHTs should aim to improve the experience of both patients and providers through human-centered design thinking principles [58]. Such a process considers the needs and perspectives of all stakeholders during the product development cycle and implementation in a health care setting. With the right design, providers can interact with DHTs more easily to gain valuable insights on their patients’ health, without compromising their existing workflow. In addition, successful implementation of DHTs in the clinical setting demands time and resources; new programs deploying DHTs should assess all the additional resources required for managing and coordinating care of patients to reduce the burden on providers.

Furthermore, providers often require hospital leadership to be supportive of a culture of innovation within their organization while weighing risks and benefits to patients and providers [38]. Therefore, organizational commitment to engaging providers at an early stage of DHT implementation by evaluating provider needs, identifying provider champions for implementing DHTs, and providing adequate training in the hospitals are critical to foster adoption.

Although not a prominent theme in this review, some studies show that the current health care policy and regulatory landscape are increasing pressure on health care organizations to provide lower-cost and higher-quality health care [59,60]. With tightening health care budgets, identifying long-term return on investment (ROI) on DHTs and establishing financial incentives through a clear reimbursement policy for providers are vital factors in increasing provider adoption. Therefore, future studies should incorporate discussions of implementation costs and ROI, in addition to examining clinical outcomes seen as a result of DHTs.

### Limitations

First, as technology and policy are evolving at a rapid pace, certain barriers and facilitators that were identified in older articles may be less relevant today. Nevertheless, some facilitators and barriers are likely to remain constant over time, such as the critical importance of integration of DHTs into clinical workflow and technology usability. Second, reporting barriers and facilitators was not the primary aim of some of the studies included in this review. Thus, a portion of the data was collected from impressions reported in discussion sections of the published studies, which includes interpretations and speculations made by the researchers involved in the studies. Finally, some of the studies included in this review provided little context on barriers and facilitators reported. In such instances, reviewers used their best judgement to determine whether the barriers or facilitators reported were best categorized as provider- or patient-related barriers or facilitators. Regardless of limitations, the themes in this review provide comprehensive evidence that could better inform and strengthen DHT development and implementation.

### Conclusions

Our findings suggest that DHT adoption for hypertension is influenced by several important factors such as integration into the clinical workflow, usability, improvements in patient outcomes, and positive impact on the patient-provider relationship. Real-world testing and incorporating feedback from both patients and providers in designing technologies will improve their overall usability. Finally, to fully realize the potential of digitally enabled hypertension management, there is a greater need to validate these technologies to provide patients and providers with reliable and accurate information on both clinical outcomes and cost effectiveness.

## Appendix

Multimedia Appendix 1 PubMed Strategy.

Multimedia Appendix 2 Summary of characteristics and authors’ analysis of the 36 studies included in the review.

## Abbreviations

AMA: American Medical Association
BP: blood pressure
CINAHL: Cumulative Index to Nursing and Allied Health Literature
DHT: digital health technology
EMBASE: Excerpta Medica database
MeSH: medical subject heading
RCT: randomized controlled trial
ROI: return on investment
UTAUT: unified theory of acceptance and use of technology
