# Supplementary material for: Provider- and Patient-Related Barriers to and Facilitators of Digital Health Technology Adoption for Hypertension Management: Scoping Review
Source: JMIR Cardio. 2019 Mar 26;3(1):e11951. doi: 10.2196/11951 (PMC6834226; doi:10.2196/11951)
Supplement: Multimedia Appendix 1 [file cardio_v3i1e11951_app1.pdf]

Appendix 1: PubMed Search Strategy

("Hypertension"[Mesh] OR hypertensi*[tiab] OR prehypertensi*[tiab] OR blood pressure[tiab])

AND

(("Mobile Applications"[Mesh] OR "Cell Phones"[Mesh] OR "Computers, Handheld"[mesh] OR ((app[tiab] OR apps[tiab] OR application*[tiab] OR technology[tiab] OR platform*[tiab] OR computer program*[tiab] OR software[tiab]) AND (smartphone*[tiab] OR phone[tiab] OR phones[tiab] OR tablet*[tiab] OR handheld*[tiab] OR iphone*[tiab] OR ipad*[tiab] OR android*[tiab])) OR mobile app*[tiab] OR mobile technolog*[tiab] OR mobile device*[tiab] OR mobile comput*[tiab] OR wearable[tiab]) OR ("Decision Support Systems, Clinical"[Mesh] OR decision support*[tiab]) OR ("Telemedicine"[mesh] OR telemedicine[tiab] OR telemonitor*[tiab] OR remote monitor*[tiab] OR televist*[tiab] OR telehealth*[tiab] OR mobile health*[tiab] OR ehealth*[tiab] OR mhealth*[tiab] OR connected health*[tiab]) OR ("Health Records, Personal"[Mesh] OR "Electronic Health Records"[Mesh] OR "Medical Records Systems, Computerized"[Mesh] OR personal health record*[tiab] OR personal medical record*[tiab] OR personally controlled health record*[tiab] OR Personal Electronic Health Record*[tiab] OR Computerized Patient Record*[tiab] OR computerized record*[tiab] OR electronic health record*[tiab] OR electronic medical record*[tiab] OR emr[tiab] OR emrs[tiab] OR phr[tiab] OR phrs[tiab] OR pchr[tiab] OR pchrs[tiab] OR ehr[tiab] OR ehrs[tiab] OR automated health record*[tiab] OR automated medical record*[tiab]))

AND

("Providers"[mesh] OR "Nurses"[mesh] OR physician*[tiab] OR nurse*[tiab] OR provider*[tiab] OR clinician*[tiab])
