# Supplementary material for: Provider- and Patient-Related Barriers to and Facilitators of Digital Health Technology Adoption for Hypertension Management: Scoping Review
Source: JMIR Cardio. 2019 Mar 26;3(1):e11951. doi: 10.2196/11951 (PMC6834226; doi:10.2196/11951)
Supplement: Multimedia Appendix 2 [file cardio_v3i1e11951_app2.pdf]

Authors, year, country	Health care setting	Study design	Study objective	Sample size	Intervention category (classified per the AMA digital health survey)	Intervention description (components of DHT used in the study)
Grant et al, 2012, United States [19]	Primary	Qualitative assessment	Assess implementation feasibility and barriers/facilitators for patient medication self-titration and software design through a small pilot study and focus groups	904	Remote monitoring for efficiency, remote monitoring and management for improved care	Hypertension management program: wireless enabled BP device combined with information management platform
Watson et al, 2012, United States [20]	Primary	RCT	Evaluate whether worksite access to an automated, Web-based, self-management program resulted in better BP control	1418	Remote monitoring and management for improved care	Home BP monitoring, most patients had an automated device with an upper arm cuff
Eklind et al, 2017, United Kingdom [21]	Specialty	Qualitative assessment	Evaluate a self-care technology to reduce hemoglobin A_{1c}, BP, and health care utilization	588	Clinical decision support	Computer-generated decision support designed to improve guideline concordant medical therapy at each visit
Bosworth et al, 2009, United States [22]	Primary	RCT	Evaluate a computer-generated decision support designed to improve	19	Remote monitoring and management for improved care	Interactive data sampling unit, which transmits keyed in data to a central computer using an

			guideline concordant medical therapy at each visit on improving BP control			automatic modem connection. The device is also equipped with 4 electrodes that record an electrocardiogram rhythm strip when the device is held firmly between the hands for 15 seconds
Shelley et al, 2011, United States [23]	Primary	Pre- and postdesign	Assess the impact of electronic clinical decision support and performance feedback on provider adherence to guideline-recommended care and BP control	297	Televisits	Telemedicine equipment consisting of a video link between the physician and the patients, supported by a system for accessing medical data via the internet
Varis et al, 2009, Finland [24]	Specialty	Qualitative assessment	Evaluate the usefulness of the DOC@HOME system for BP monitoring and control in Finnish hypertensive patients	4251	Clinical decision support, point-of-care	Previsit electronic journals containing electronic health record–based medication, allergies, diabetes or health maintenance personal history, and family history topics to share with a provider
Abdullah et al, 2016, Malaysia [25]	Primary	Qualitative assessment	Assess patient's acceptance of a BP	1102	Clinical decision support	Web-based individualized

			telemonitoring service			vascular tracking and advice-decision support system outlining the top vascular risk factors
Vedanthan et al, 2015, Kenya [26]	Specialty	Usability pilot	Conduct usability and feasibility testing of a tablet-based decision support and integrated record-keeping tool for providers	3636	Clinical decision support	A tailored multicomponent clinical decision support system, which included a BP alert, a hypertension order set, a template, and clinical reminders
DeAlleaume et al, 2015, United States [27]	Primary	Pre- and postdesign	Evaluate the impact of the home BP management system on hypertension control	16	Remote monitoring and management for improved care, clinical decision support	System included alerts, order sets, templates, clinical reminder algorithms, and provider performance feedback
Czaja et al, 2014, United States [28]	Primary	Usability pilot	Identify problems associated with the implementation and functioning of a home telehealth system and the participants' experience in using the system	316	Remote monitoring and management for improved care	Telephone surveys of nonparticipants in a home BP monitoring program, which supplied a complimentary automated BP cuff and utilized a centralized reporting system

Kim et al, 2015, United States [29]	Primary	RCT	Determine whether there is a difference in BP lowering per Web-based remote patient monitoring with or without remote physician care	296	Remote monitoring and management for improved care	Wireless home BP cuff and telemedicine device
North et al, 2015, United States [30]	Primary	Qualitative assessment	Examine patient-generated secure messages containing self-monitored BP content and study how their providers responded to these messages	60	Remote monitoring for efficiency, remote monitoring and management for improved care	BP monitoring platform where automatic alerts were generated by the central database server using predefined algorithms and patients were subsequently contacted by the physician
Sublet et al, 2015, United States [31]	Primary	Qualitative assessment	Collect data from providers and patients on the expectations of use of their technical equipment	186	Televisits	Community based telehealth model for hypertension
Juan Frias, 2016, United States [32]	Primary	RCT	Evaluate the use of an intervention consisting of a sensor-enabled medicine, a wearable sensor, patient app, and provider portal, to	70	Patient engagement	Web-based system for patient antihypertensive medication self-titration on the basis of primary care physician-defined pathways

			optimize BP and cholesterol in patients with uncontrolled hypertension and diabetes mellitus			
Ideal Life, 2008, United States [33]	N/A	N/A	Analyze patients using real-time readings on the hypertension management program for 6 months to improve BP control	404	Patient engagement, tools providing consumer access to clinical data	Automated, Web-based, self-management program with worksite access to an onsite BP cuff
Kim et al, 2015, South Korea [34]	Primary and specialty	Qualitative assessment	To identify the factors that contribute to successful telemedicine service	124	Remote monitoring and management for improved care, patient engagement	Telehealth approach required patients to take home BP readings and text them to a secure server for immediate automatic analysis and individual health care professional review
Neumann et al, 2011, Germany [35]	Primary	RCT	Examine the effect of telemedicine BP monitoring and the telemedicine BP monitoring–derived therapeutic action on the quality of BP control in a defined	591	Televisits	Home BP monitor and telemedicine device to transmit BP measurements

			short study period of 3 months			
Kressin et al, 2016, United States [36]	Primary	RCT	Evaluate the effectiveness of 2 provider-focused interventions (an electronic medical record reminder and a clinician focused communication skills training intervention) to improve hypertension care and outcomes	401	Remote monitoring and management for improved care	Telemonitoring and supervision by usual primary care clinicians of home self-measured BP and optional patient decision support
Chen et al, 2011, Taiwan [37]	Primary	Qualitative assessment	Evaluate acceptance of the community-based telehealth model on hypertension in Taipei	45	Remote monitoring and management for improved care	Telemonitoring system for BP with a Web-based portal
Cottrell et al, 2012, United Kingdom [38]	Primary	Qualitative assessment	Determine the patient experience of using a simple telehealth strategy to manage hypertension in adults	27	Remote monitoring and management for improved care	Evidence-based nurse-delivered self-management phone intervention to facilitate hypertension management
Wakefield et al, 2014, United States [39]	Primary	RCT	Evaluate the effectiveness of short-term targeted use of data transmission from in-home	132	Remote monitoring for efficiency, remote monitoring and management for improved care	Personalized physician learning intervention with electronic health record in which personalized

			devices (blood glucose meter and BP machine) to the clinic on treatment outcomes in patients with diabetes who had either out-of-range hemoglobin A_{1c} or systolic BP measurements			physician learning cases were assigned to each physician on the basis of observed patterns of care in the electronic health record in the previous year
Frias et al, 2017, United States [40]	Primary	RCT	Assess the impact on clinic-measured BP and hemoglobin A_{1c} through a digital medicine offering that measures medication ingestion adherence, physical activity, and rest using digital medicines (medication taken with ingestible sensor),wearable sensor patches, and a mobile device app	34	Remote monitoring and management for improved care	Telehealth system consisting of 3 devices: a BP measuring unit, a bodyweight scale, and a wireless unit to transmit the data to a website through a telephone line
Hanley et al, 2013, United Kingdom [41]	Primary	Qualitative assessment	Explore the experiences of patients and professionals taking part in an	108	Remote monitoring and management for improved care	Home telemonitoring for glycemic and BP control where short-term targeted use of

			RCT of remote monitoring supported by primary care			data transmission from in-home devices to the clinic on treatment outcomes in patients with diabetes who had either out-of-range hemoglobin A_{1c} or systolic BP measurements
O'Connor et al, 2014, United States [42]	Primary	RCT	Assess the impact of personalized physician learning interventions using simulated learning cases on control of hypertension and dyslipidemia in primary care settings.	138	Remote monitoring for efficiency, televisits	Structured physician-directed, nurse-managed telephone support with BP telemonitoring
Mann et al, 2015, Canada [43]	Primary	Qualitative assessment	Develop a real-time electronic medical record tool on the basis of the 2014 Canadian hypertension education program guidelines	13	Clinical decision support	A tablet-based decision support and integrated record-keeping tool
Wang et al, 2012, United States [44]	Primary	RCT	Evaluate the impact of home BP monitoring and telephone-based interventions on patients with	212	Patient engagement	Secure messages on a patient portal that allowed patients to asynchronously communicate with

			inadequate BP control			their health care teams
Nilsson et al, 2009, Sweden [45]	Primary	Nonrandomized trial	Evaluate the feasibility and quality of uncomplicated hypertension care in rural areas (treatment in practice). A nurse-led surgery with doctor support by telemedicine was compared with a surgery with face-to-face consultations between providers and patients	3578	Remote monitoring and management for improved care	Home BP monitoring program: one size cuff, participants had to monitor their BP once a day at a different time every day.
Bernocchi et al, 2014, Italy [46]	Primary	Nonrandomized trial	Evaluate the effectiveness of a structured physician-nurse approach supported by remote BP telemonitoring in patients with uncontrolled hypertension	374	Remote monitoring for efficiency	Communication technologies, remote monitoring coupled with physician care
Crowley et al, 2011, United States [47]	Primary	RCT	Evaluate physician reactions to elevated home BP readings in a	10,000	Remote monitoring and management for improved care, televisits	Remote monitoring devices, wearables, video consultations with providers

			telemanagement trial			
Holbrook et al, 2011, Canada [48]	Primary	RCT	Evaluate if a clinical decision support system improves patient care and patient health outcomes	57	Televisits	Self-administered questionnaire to better understand how telemedicine for improved BP control affects care
Huff et al, 2011, United States [49]	Specialty	Qualitative assessment	Evaluate the use of monitors, perceived benefit, communication with providers, and possible barriers.	1084	Point-of-care	Electronic health record tool developed from evidence-based guidelines
McKinstry et al, 2013, United Kingdom [9]	Primary	RCT	Determine if an intervention consisting of telemonitoring and supervision by usual primary care clinicians of home self-measured BP and optional patient decision support leads to clinically important reductions in daytime systolic and diastolic ambulatory BP in patients with uncontrolled BP	8866	Clinical decision support	Electronic health record reminder for hypertension care and a clinician focused communication skills training intervention to improve communication with patients about BP and antihypertensive medication adherence
Millery et al, 2011, United States [50]	Primary	Qualitative assessment	Examine the implementation of an electronic	17	Remote monitoring and management for improved care	BP telemonitoring service delivered in primary care

			health record–based intervention to improve quality of hypertension care in community health centers and use qualitative analysis to explain how different components of the intervention contributed to positive patient-level outcomes			
Shaw et al, 2013, United States [51]	Primary	Qualitative assessment	Determine what is the level of organizational readiness to implement the intervention and specific facilitators, barriers, and contextual factors that may affect organizational readiness to change	109	Remote monitoring for efficiency, remote monitoring and management for improved care	Digital health offering consisting of sensor-enabled medications, a wearable sensor, patient app, and provider portal
Wald et al, 2010, United States [52]	Primary	RCT	Evaluate the patient and provider implementation experience through analysis of electronic journal	109	Patient engagement	Ingestible sensors, wearable sensors, and mobile app to track physical activity

			usage and survey data			
Logan et al, 2008, Canada [14]	Primary	Qualitative assessment	Assess primary care physician attitudes to home BP monitoring	93	Remote monitoring and management for improved care	An interface for both patients and clinicians and a personal health channel on participant's television or a tablet or smartphone app
